# Cardiac fiber tracking on super high-resolution CT images: a comparative study

**DOI:** 10.1117/1.JMI.7.2.026001

**Published:** 2020-03-11

**Authors:** Hirohisa Oda, Holger R. Roth, Takaaki Sugino, Naoki Sunaguchi, Noriko Usami, Masahiro Oda, Daisuke Shimao, Shu Ichihara, Tetsuya Yuasa, Masami Ando, Toshiaki Akita, Yuji Narita, Kensaku Mori

**Affiliations:** a Nagoya University, Graduate School of Informatics, Nagoya, Japan; b Nagoya University Graduate School of Medicine, Department of Radiological and Medical Laboratory Sciences, Nagoya, Japan; c Nagoya University School of Medicine, Department of Tissue Engineering, Nagoya, Japan; d Hokkaido University of Science, Department of Radiological Technology, Sapporo, Japan; e Nagoya Medical Center, Clinical Research Center, Department of Pathology, Nagoya, Japan; f Yamagata University, Graduate School of Engineering and Science, Yamagata, Japan; g Tokyo University of Science, Research Institute of Science and Technology, Tokyo, Japan; h Nagoya University, Information Technology Center, Nagoya, Japan; i National Institute of Informatics, Research Center for Medical Bigdata, Tokyo, Japan

**Keywords:** high-resolution cardiac imaging, heart staining protocols, microtomography, fine anatomical structure analysis

## Abstract

**Purpose:** High-resolution cardiac imaging and fiber analysis methods are required to understand cardiac anatomy. Although refraction-contrast x-ray CT (RCT) has high soft tissue contrast, it cannot be commonly used because it requires a synchrotron system. Microfocus x-ray CT (μCT) is another commercially available imaging modality.

**Approach:** We evaluate the usefulness of μCT for analyzing fibers by quantitatively and objectively comparing the results with RCT. To do so, we scanned a rabbit heart by both modalities with our original protocol of prepared materials and compared their image-based analysis results, including fiber orientation estimation and fiber tracking.

**Results:** Fiber orientations estimated by two modalities were closely resembled under the correlation coefficient of 0.63. Tracked fibers from both modalities matched well the anatomical knowledge that fiber orientations are different inside and outside of the left ventricle. However, the μCT volume caused incorrect tracking around the boundaries caused by stitching scanning.

**Conclusions:** Our experimental results demonstrated that μCT scanning can be used for cardiac fiber analysis, although further investigation is required in the differences of fiber analysis results on RCT and μCT.

## 1 Introduction

Deep understanding of the cardiac fiber structure in the left ventricle (LV) is required to understand cardiac anatomy and such diseases as heart failure. 6.5 million people experienced heart failure between 2011 and 2014 in the United States.^1^ Although the fiber structure may also be changed by heart failure, the details have not been clearly investigated yet. High-resolution cardiac imaging and analysis methods in three-dimensional (3-D) space are needed.

Diffusion tensor magnetic resonance imaging (DT-MRI) is well-known for analyzing cardiac fiber structure.^2^^–^^4^ With DT-MRI, we estimate the fiber orientation at a point as the orientation with the strongest diffusion of water molecules. However, its resolution is inadequate. For instance, Helm et al.^3^ used a 1.5-T CV/I MRI scanner (General Electric) whose resolutions were 300, 300, and 800  μm for each of three axes. Histopathological images have also been used^5^^,^^6^ for cardiac imaging with much higher resolution than DT-MRI. However, precise reconstruction of the heart’s stacked section images is complicated due to the tissue damage caused by cutting the sections and the banana problem.^7^ 3-D analysis from a heart’s histopathological stacks is very difficult.

We explored two alternate scanning modalities: refraction-contrast x-ray CT (RCT) and microfocus x-ray CT (μCT). RCT^8^^,^^9^ is a 3-D imaging modality that is one type of phase-contrast CT scanning based on observing the refraction of x-rays. It has very high soft tissue contrast, even for cardiac fibers. However, RCT is not commercially available and cannot be utilized publicly because it requires a synchrotron system.

μCT is a commercially available 3-D imaging modality. In general, scanning is done by observing the absorption of x-rays that run through target objects. Resolution, contrast, and image size vary, as do their price ranges. Some scanners, which also observe phase shift, have very high resolution; SCYSCAN 1727 (Bruker) has the highest: 0.35-μm resolution. We utilize a relatively low-end type of scanner, inspeXio SMX-90CT Plus (Shimadzu, Japan), which only observes x-ray absorption; its highest resolution is around 5  μm. Nevertheless, although cardiac fibers can be observed on the μCT volumetric images (volumes) produced by this scanner, their contrast is not as clear as RCT volumes.

In this paper, we first describe our fiber analysis methods from the RCT or μCT volumes of the heart. Then, we analyze how μCT produces proper results by qualitatively and quantitatively comparing it with RCT. Fiber analysis consists of the estimation of orientation and tracking fibers and compares the results from a μCT volume with those of an RCT volume. We prepared a heart specimen with our original protocols and scanned it with RCT and μCT and registered their volumes. Using these registered RCT and μCT volumes, we compared the fiber orientation estimation results on a slice to check quantitatively whether the μCT volume produced similar fiber orientation estimation results as the RCT volume. We tracked the fibers to investigate whether fiber orientation can be estimated well on the μCT volume in the entire LV. Our experimental results demonstrated that μCT scanning can be used for cardiac fiber analysis, although further investigation is required of the differences of the fiber analysis results on RCT and μCT. This paper is an extended version of our 2019 SPIE Proceedings paper.^10^

## 2 Fiber Analysis Method

### 2.1 Overview

Our fiber analysis method consists of the following two schemes: (1) estimation of fiber orientation and (2) fiber tracking.

We did scheme (1) for each voxel in the input CT volume to estimate the fiber orientation around the voxels to quantitatively analyze the fiber orientation statistics.

We did scheme (2) on the entire CT volume to produce trajectories that follow the fibers. Scheme (1) must be performed during the tracking process. The results of scheme (2) are useful for qualitatively visualizing how fibers flow in the entire LV.

### 2.2 Fiber Orientation Estimation

Structure tensor (ST) analysis is commonly used for estimating the cardiac fiber orientation in μCT volumes.^11^^,^^12^ First, for each volume, we apply a Gaussian smoothing filter with standard deviation $\sigma_{P}$ to smooth the intensity gradients and empirically set $\sigma_{P}=20\;\mu m$.

ST T(x) at voxel x is defined:

$$T(x)=\underset{x^{\prime }\in N}{\sum }w(\sigma_{T},\|x-x^{\prime }\|)g(x^{\prime })g^{T}(x^{\prime }),$$ (1)

where N is a set of the neighboring voxels around x, $x^{\prime }$ is one of the voxels in N, $w(\sigma_{T},\|x-x^{\prime }\|)$ is the Gaussian weight with standard deviation $\sigma_{T}$ and distance $\left\|x-x^{\prime }\right\|$ from the center, and $g(x^{\prime })$ is a local intensity gradient vector around $x^{\prime }$. T(x) can be written as a 3×3 matrix. The eigenvector of T(x), which corresponds to smallest eigenvalue f(x), is assumed to be a direction of the fiber orientation at x, which has the smallest intensity changes around x. We set $\sigma_{T}$ to 400  μm.

### 2.3 Fiber Tracking

We randomly generated N initial points in the mask of the LV region. From each initial point, fiber tracking was done by an iterative process. First (iteration k=0), we estimated fiber direction vector ${f(x}_{0})$ at each initial point $x_{0}\in R^{3}$ using the ST analysis described in Sec. 2.2. Since the fibers are running in both directions, ${f(x}_{0})$ and $-{f(x}_{0})$, fiber tracking was also performed for both directions. We calculated the endpoint coordinates of the trajectories at the k’th iteration (iteration k>0):

$$x_{k}=x_{k-1}+sf(x_{k-1}),\;x_{-k}=x_{-(k-1)}-sf[x_{-(k-1)}],$$ (2)

where s represents the step size, $f(x_{k-1})$ represents the orientation vector at $x_{k-1}$, and $f[x_{-(k-1)}]$ represents the orientation vector at $x_{-(k-1)}$. We terminated the tracking for each direction when $x_{k}$ or $x_{-k}$ was outside the LV mask, or index k of the iterations reached $k_{\max}$. We set the parameters to $n_{i}=1000$, s=4 voxels, and $k_{\max}=1000$. The trajectories, which were tracked from all the initial points, are output.

## 3 Materials

### 3.1 RCT and μCT Scanning

RCT and μCT volumes of a rabbit heart were obtained by the following sequence: (1) harvesting a heart, (2) ethanol fixation, (3) RCT scanning, (4) contrast enhancement, (5) rinse, and (6) μCT scanning. Fixation was performed once using ethanol. $I_{2}\mathrm{KI}$ was used for the contrast enhancement for the μCT scanning. Ethanol was used again in preparation for the μCT scanning for rinsing excess $I_{2}\mathrm{KI}$ to reduce the artifact.

**Fig. 1:**
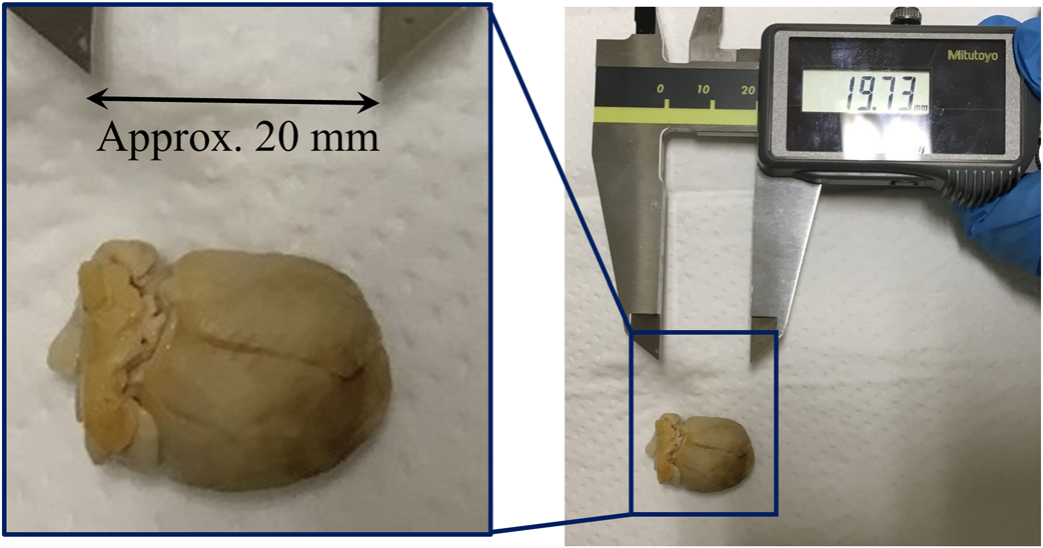
Rabbit heart: longest axis is about 20 mm.

**Fig. 2:**
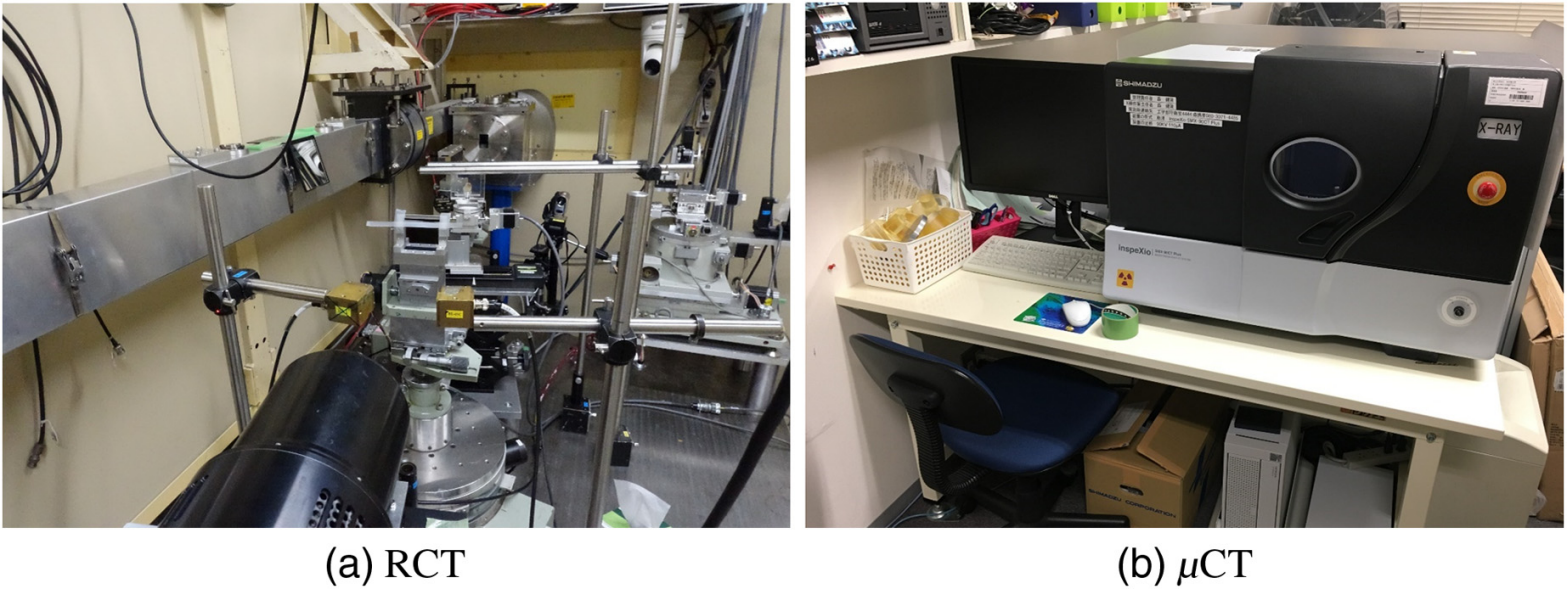
Machines that scanned rabbit heart in Fig. 1: (a) RCT and (b) μCT.

**Fig. 3:**
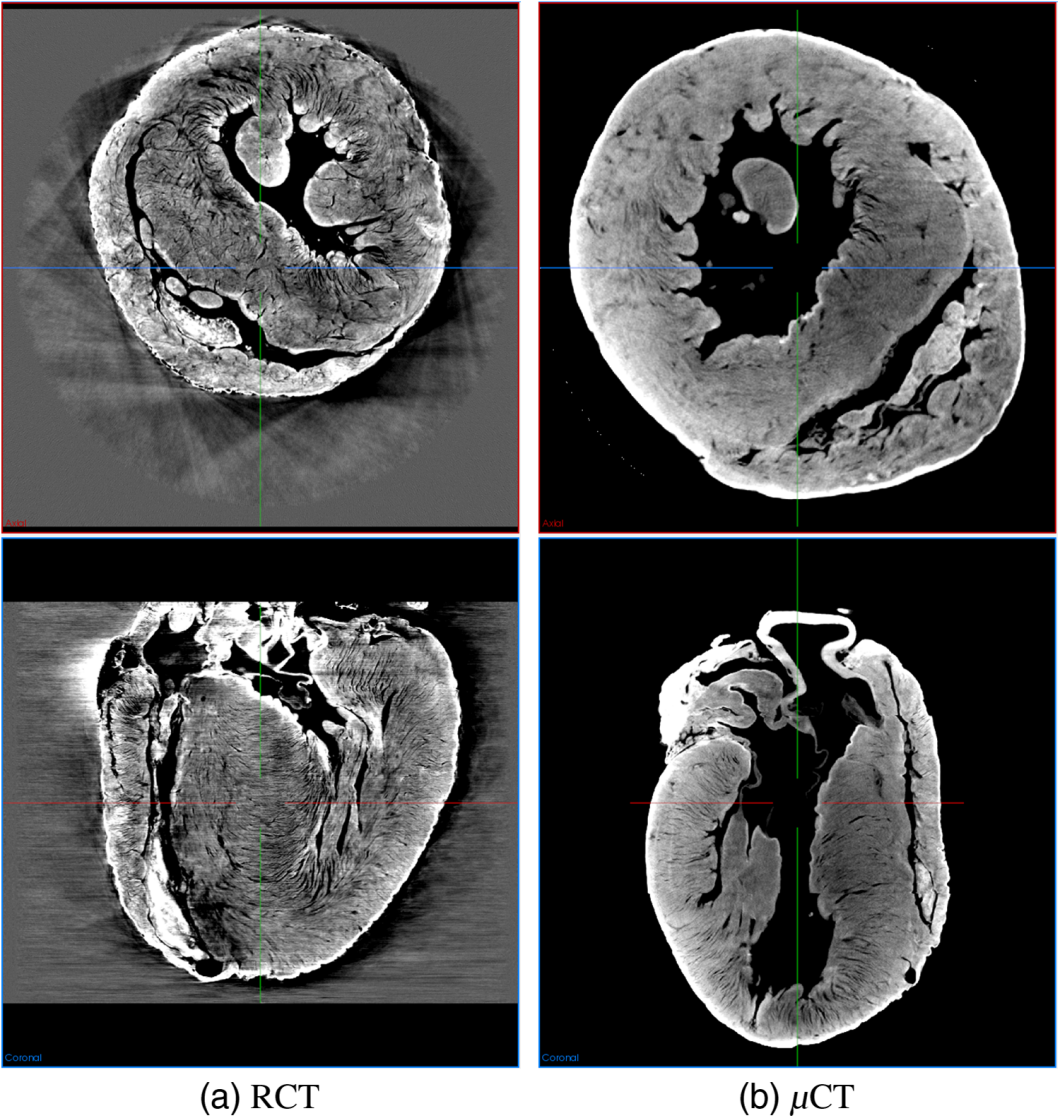
Axial and coronal slices: (a) RCT and (b) μCT volumes. Fibers on RCT volume look clearer than those of μCT volume. Registration is required for comparison due to different heart positions.

The following are the specimen preparation and scanning procedures. We scanned one μCT and one RCT volume of a rabbit heart (Fig. 1) under the IRB approval of Nagoya University. We harvested the heart of a Japanese white rabbit (10-week-old male) just after euthanasia with a KCl injection into the aortic arch and obtained a heart specimen. The following is the RCT scanning procedure: (1) ethanol fixation: the heart was fixated with an 80% ethanol water solution since ethanol fixation effectively improves the tissue contrast better than formalin fixation for the other phase-contrast imagings of hearts.^13^ (2) RCT scanning: RCT scanning was performed using the synchrotron system developed by Ando et al.’s group [Fig. 2(a)] at the High Energy Accelerator Research Organization (KEK) (Japan).^14^ The synchrotron system used for RCT scanning cost about 177 million USD (1 USD = 110 JPY).^15^ The RCT scanning specifications are listed in Table 1. Axial and coronal slices of the RCT volume are shown in Fig. 3(a).

**Table 1:** RCT scanning specifications.

Item	Value
Location for scanning	Photon Factory, High Energy Accelerator Research Organization (Tsukuba, Japan)
Camera	VHR 16 M (Photonics Science)
X-ray optical system	X-ray dark field imaging
Resolution	$15\times 15\times 15\;\mu m^{3}/\text{voxel}$
Volume size	1600×1600×1240 voxels
X-ray energy	19.8 keV

After RCT scanning, we scanned the same heart specimen in the following manner. We introduced an additional staining process for μCT scanning. (1) Contrast enhancement: We stained the rabbit heart with a 7.5% $I_{2}\mathrm{KI}$ solution for one day. (2) Rinse: the heart was briefly rinsed in an 80% ethanol solution. (3) μCT scanning. Table 2 shows the scanning specification. Our scanner’s field of view (FOV) was limited: 1024×1024×548  voxels at $17\times 17\times 17\;\mu m^{3}/\text{voxel}$ resolution. It has a stitch-scanning mode to cover larger FOVs. We used this feature to cover the entire heart (three consecutive scans), although not every volume was aligned well in the stitching mode. Furthermore, ring artifacts on the μCT volume were quite obvious. We used TomoPy^16^ to reduce the ring artifacts, which are commonly observed on μCT volumes. Examples of the axial and coronal slices of the μCT volume are shown in Fig. 3(b).

**Table 2:** Specifications of μCT scanning.

Item	Value
Location for scanning	Nagoya University (Nagoya, Japan)
Scanner	inspeXio SMX-90CT Plus (Shimadzu)
Resolution	$17\times 17\times 17\;\mu m^{3}/\mathrm{voxel}$
Volume size	1024×1024×1627 voxels
# of divided-scanning parts	4
Tube voltage	90 kVp
Tube current	110 μA
# of x-ray projection	1200
# of projections for each angle	12

This work used a desktop-type μCT scanner, inspeXio SMX-90CT Plus (Shimadzu, Japan) [Fig. 2(b)], which is a low-end, desktop type. Its catalog price is ∼236,000 USD (1  USD=110  JPY). Ethanol fixation^13^ is also suitable for μCT scanning in combination with contrast enhancement. Other μCT cardiac imaging works^12^^,^^17^ use high-end, much more expensive μCT scanners than ours. In those works, contrast enhancement continued for several days by staining the specimens in an iodine-potassium iodide ($I_{2}\mathrm{KI}$) solution. For instance, one trial by Stephenson et al.^17^ stained a rabbit heart in a 7.5% $I_{2}\mathrm{KI}$ solution for 3 days with the Metris X-Tec custom 320-kV bay system with 155-kV tube voltage and 150-μA tube current. However, directly using the same protocols as these Refs. 12 and 17 for our scanner caused artifacts since our scanner has lower x-ray energy.

RCT has superior soft tissue contrast to μCT. This means that RCT can depict different soft tissues in different intensities although μCT depicts such soft tissues in the same intensities. Phase-contrast x-ray imaging including RCT has been developed for better soft tissue contrast.

### 3.2 Registration of μCT and RCT Volumes

**Fig. 4:**
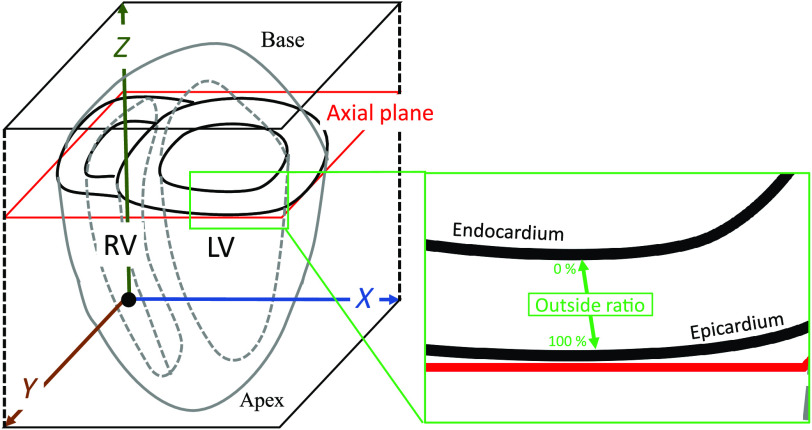
Coordinate system and position of ventricles: Axial planes (x−y plane) cut axis along base and apex into rounds. On axial planes, RV is shown on left of LV. Outside ratio is illustrated in magnified part. Outside ratio becomes 0% at endocardium side and 100% at epicardium side.

To compare the fiber analysis results, we registered the RCT volume as μCT. The heart’s μCT and RCT volumes were cropped and rotated manually using the MITK Workbench 2016.11.^18^ The LV is entirely covered with a slight margin around it and roughly aligned between the two volumes whose size and resolution were adjusted into 900×980×1080 and 18×18×18  μm/voxels respectively. The coordinate system of these volumes is shown in Fig. 4. Since the parts of the surrounding regions such as RV were also included, the processing target region was specified by masking. The mask of the LV region (LV mask) was segmented semiautomatically using the MITK Workbench 2016.11^18^ on the μCT volume. Then, we applied nonrigid registration to the RCT volume to align it with the μCT volume. We used deedsBCV, which is open-source software published by Heinrich et al.^19^

**Fig. 5:**
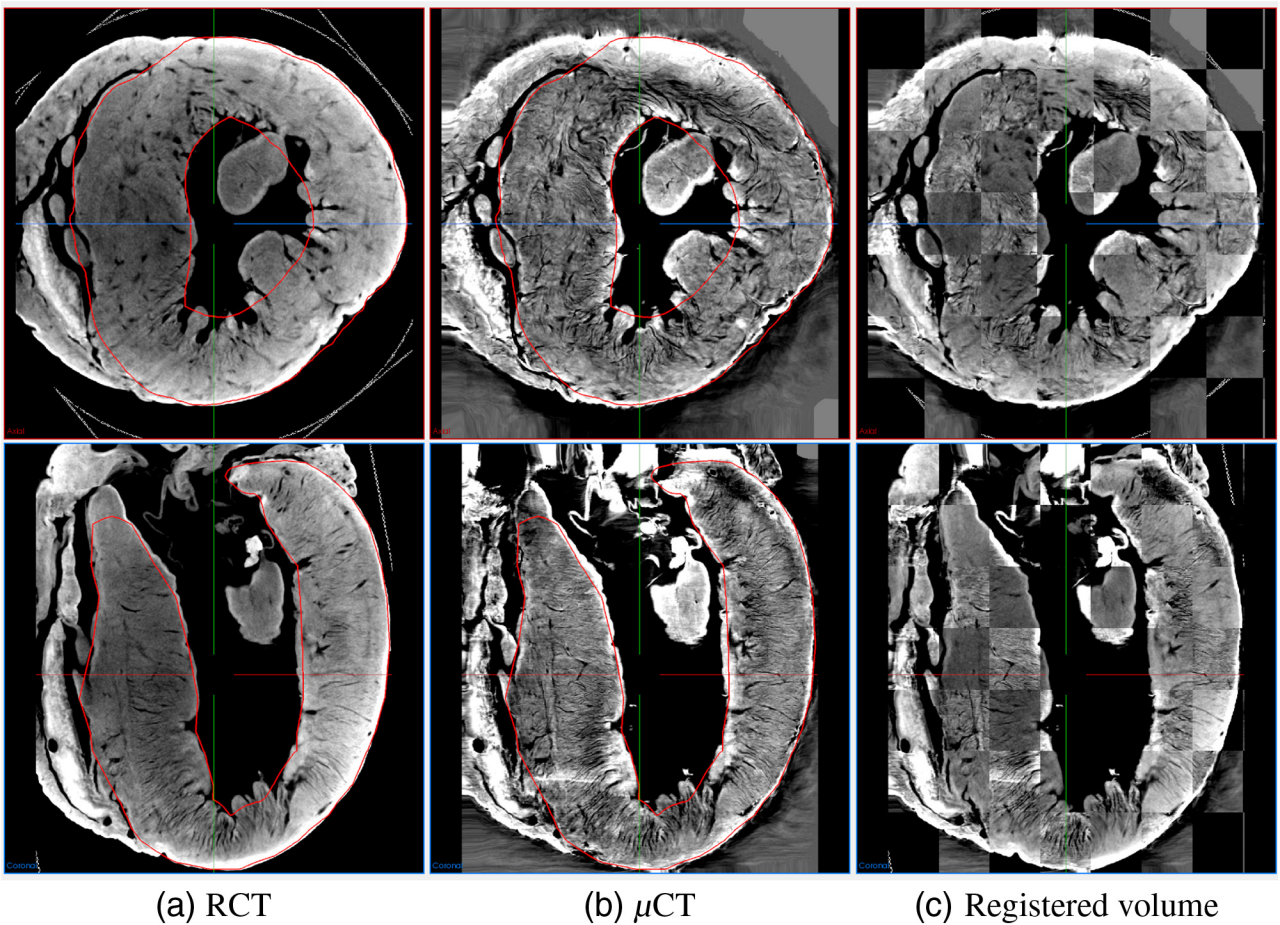
Axial and coronal slices of registered volumes: Registration results of (a) RCT and (b) μCT with LV mask (red line); (c) checkerboard-like scheme visualization of these volumes.

Figures 5(a) and 5(b) show the axial and coronal slices of the registration results. In Fig. 5(c), the axial slices of the two registered volumes are shown as one figure after being merged to resemble a checkerboard. Clearly, the RCT volume was successfully registered to the μCT volume. As shown in Fig. 5(c), the boundaries of the LV and the image patterns shown in both volumes were successfully aligned.

## 4 Experimental Setup

### 4.1 Overview

We evaluated how our fiber analysis method produced precise results from the μCT volume by comparing them with the RCT volume results. We performed fiber tracking for each registered volume to compare the tracking results obtained from the RCT and μCT volumes. We analyzed the fiber orientation statistics on multiple axial slices. Detailed analysis was conducted on one of those axial slices around the central part of the LV and focused on fiber orientations. The 3-D visualization of the fibers was performed by fiber tracking (Sec. 2.3).

### 4.2 Fiber Orientation Statistics

#### 4.2.1 Definition of outside ratio

Anatomical studies^5^^,^^20^ clarified that inside and outside of the LV tends to have different fiber orientations. Therefore, analyzing the fiber orientations may produce different results that correspond to their respective positions inside and outside the LV. We define the outside ratio measure based on whether each sample point is represented as nearer the outside of the LV wall than inside it. The outside ratio becomes 0% at the endocardium side and 100% at the epicardium side, as illustrated in the magnified part of Fig. 4.

From the center point of the LV region on an axial slice, we performed radial searches to eight angles on an axial slice. On each search, we obtained a set of sample points whose outside ratios were 10%, 20%, ⋯, or 90%. On each sample point, we individually estimated the fiber orientation from the RCT and μCT volumes, where the axial slices cut the heart orthogonally to its longest axis (Fig. 4).

#### 4.2.2 Angle difference of μCT from RCT

**Fig. 6:**
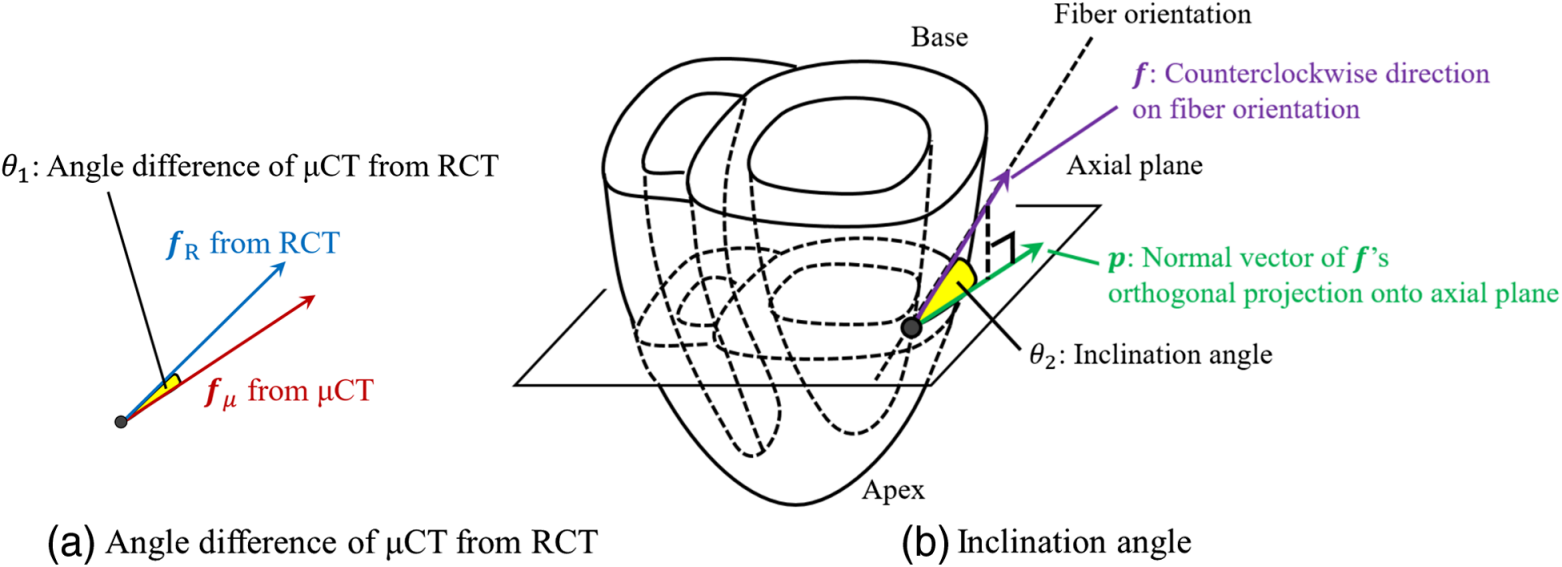
Definitions of angles: (a) angle difference of μCT from RCT $\theta_{1}$ and (b) inclination angle $\theta_{2}$.

We define the angle difference of μCT from RCT $\theta_{1}$:

$$\theta_{1}=\cos^{-1}\{f^{\mu }(x)\cdot f^{R}(x)\}\;(0\leq \theta_{1}\leq \pi ),$$ (3)

where $f^{\mu }(x)$ and $f^{R}(x)$ represent the unit vectors of the fiber orientations estimated from the μCT and RCT volumes [Fig. 6(a)], respectively. Assuming the orientation from RCT is the ground-truth, the angle difference of μCT from RCT represents the estimation error on μCT.

To evaluate how the fiber orientations estimated from μCT volumes are different from those of the RCT, we computed the average and standard deviations of the angle difference of μCT from RCT at 100-slice intervals and plotted them on a graph. We also visualized the angle differences of μCT from RCT on sample points on an axial slice around the central area.

#### 4.2.3 Inclination angle

Inclination angle $\theta_{2}$ follows anatomical studies. Streeter et al.^5^ defined fiber angle α and showed that it becomes positive inside and negative outside the LV. Our definition of inclination angle resembles their definition, which can be computed in 3-D volumes. As shown in Fig. 6(b), the inclination angle is defined as

$$\theta_{2}=\cos^{-1}\{f(x)\cdot p\}\;(-\pi <\theta_{2}\leq \pi ),$$ (4)

where f(x) represents the estimated fiber orientation. $p=\frac{p[f(x)]}{\left\|p[f(x)]\right\|}$ represents a unit vector on the axial plane, written by orthographic projection p[f(x)] of f(x) onto the axial plane.

We visualized the angle difference of μCT from RCT of each sample point on an axial slice around the central area. We drew a scatter plot of the inclination angles computed from μCT and RCT and verified the statistical significance of the correspondence. We also observed the correlation between the outside ratio and the inclination angle for each volume. Their significant correlations suggest that the results follow the anatomical knowledge that the fiber orientations are different inside and outside LV.

### 4.3 3-D Visualization of Fibers

We performed 3-D visualization using open-source software ParaView 5.3.0^21^ for each registered volume to qualitatively compare the fiber trajectories from the RCT and μCT volumes in the entire LV. All the points of the trajectories were colored to show the inclination angle. We showed all the tracking results. We trimmed them and showed whether for the sagittal slices, the tracking was done properly in the entire LV. Since ParaView crashed when we directly opened the RCT or μCT volumes, we downsampled these volumes twice by cubic interpolation before opening them.

## 5 Results

### 5.1 Fiber Orientation Statistics

**Fig. 7:**
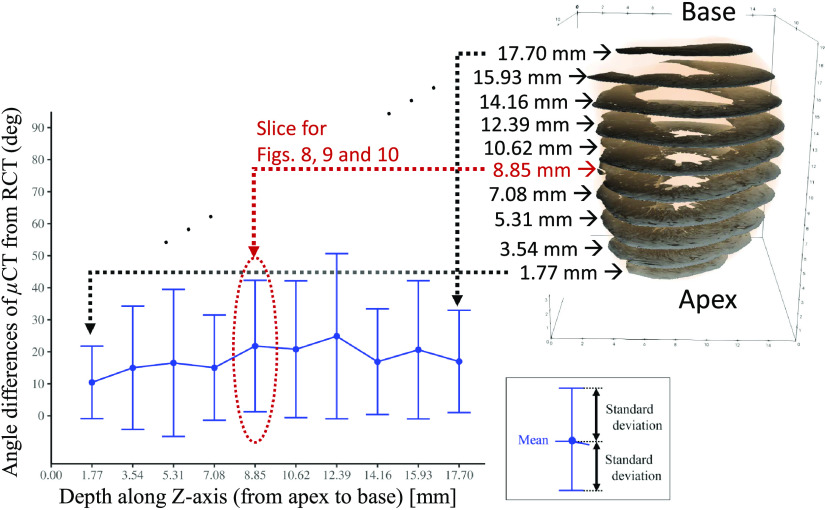
Mean and standard deviation of angle differences of μCT from RCT at sample points on each axial slice: target axial slices were selected at 100-slice intervals along Z-axis (longest axis from apex to base) of RCT and μCT volumes. Each point on graph shows mean angle differences of μCT from RCT of a sample point on slice, and error bars represent standard deviation. Sample points on each slice were defined by a radial search scheme, explained in Sec. 4.2. Results do not greatly vary throughout the entire LV. Slice located at depth 8.85 mm is used for further evaluation in Figs. 8–10 and indicated by arrow.

**Fig. 8:**
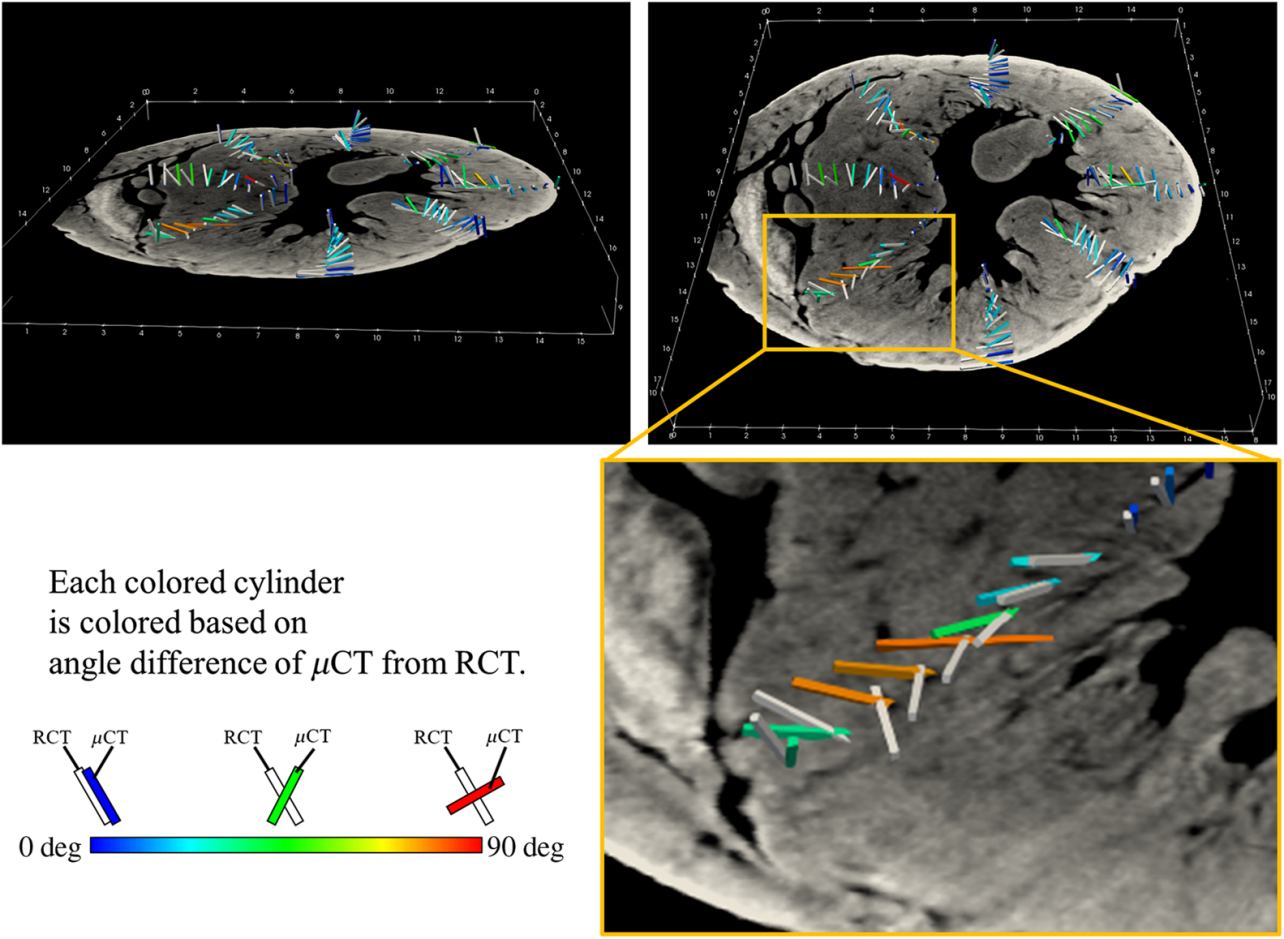
Angle differences of μCT from RCT on manually selected slice (depth = 8.85 mm, see Fig. 7): colored cylinders show fiber orientations estimated from μCT volume, colored based on angle difference of μCT from RCT. Fiber orientations estimated from RCT volume are also shown as white cylinders.

Figure 7 shows the mean and standard deviation of the angle differences of μCT from RCT on axial slices throughout the LV, most of which had mean angle differences of μCT from RCT around 20 deg. For instance, their mean and standard deviations were 21.8±20.5  deg on an axial slice around the central part. Figure 8 shows the angle differences of μCT from RCT on a manually selected slice (depth=8.85 mm. see Fig. 7). In Fig. 8, fiber orientations at a sample point are represented as two cylinders. A white cylinder shows the fiber orientation estimated from the RCT volume. The colored cylinder shows fiber orientation estimated from the μCT volume, colored based on their angle difference of μCT from RCT.

**Fig. 9:**
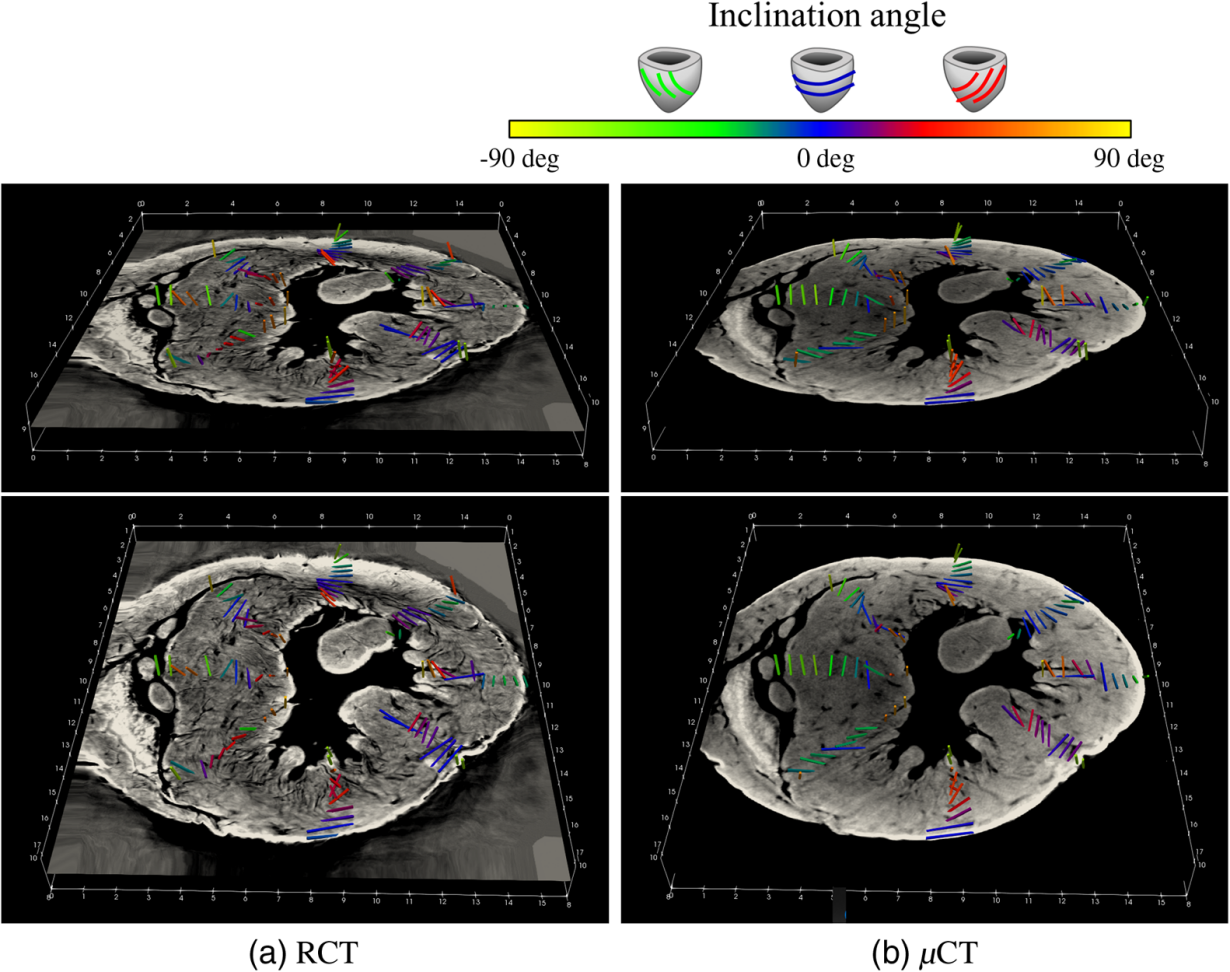
Fiber orientations on manually selected slice (depth = 8.85 mm. see Fig. 7) with coloring based on inclination angle: (a) RCT and (b) μCT. Cylinders show estimated fiber orientations; colors represent inclination angles.

Figure 9 also shows the estimated fiber orientations. Cylinders show estimated fiber orientation, colored based on their inclination angles.

**Fig. 10:**
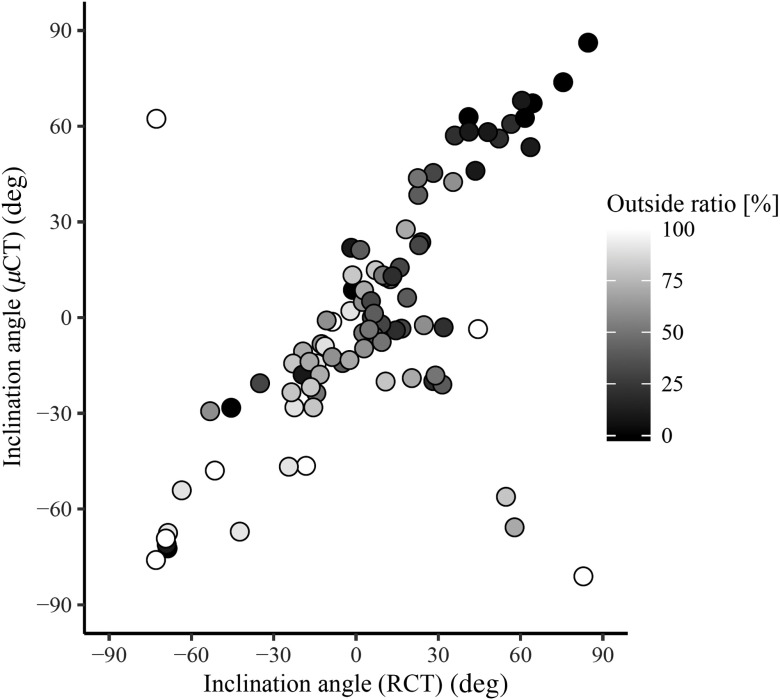
Relationship of inclination angles measured in RCT and μCT volumes. We manually selected slice (depth = 8.85 mm, see Fig. 7) and plot inclination angles measured on selected slice in this figure. Each circle is gray-scale coded based on outside ratio. Positive correlation is clearly observed between inclination angles estimated from RCT and μCT volumes. We can also observe positive inclination angles in outside area (epicardium area).

The relationship of the inclination angles measured in the RCT and μCT volumes is shown in Fig. 10. Each circle in Fig. 10 is gray-scale coded based on the outside ratio. The inclination angles estimated from the RCT and μCT volumes had a correlation coefficient (CC) of 0.63. No significant difference was observed by Spearman’s significant test: $p<2.2\times 10^{-16}$. This shows μCT produced fiber analysis results that resembled those of RCT. The inclination angles of RCT and the outside ratio also show a significant correlation: $p=2.4\times 10^{-6}$ with a CC of −0.48. Those of the μCT and the outside ratio are $p=1.2\times 10^{-7}$ and showed a correlation with a CC of −0.53.

### 5.2 3-D Visualization of Fibers

**Fig. 11:**
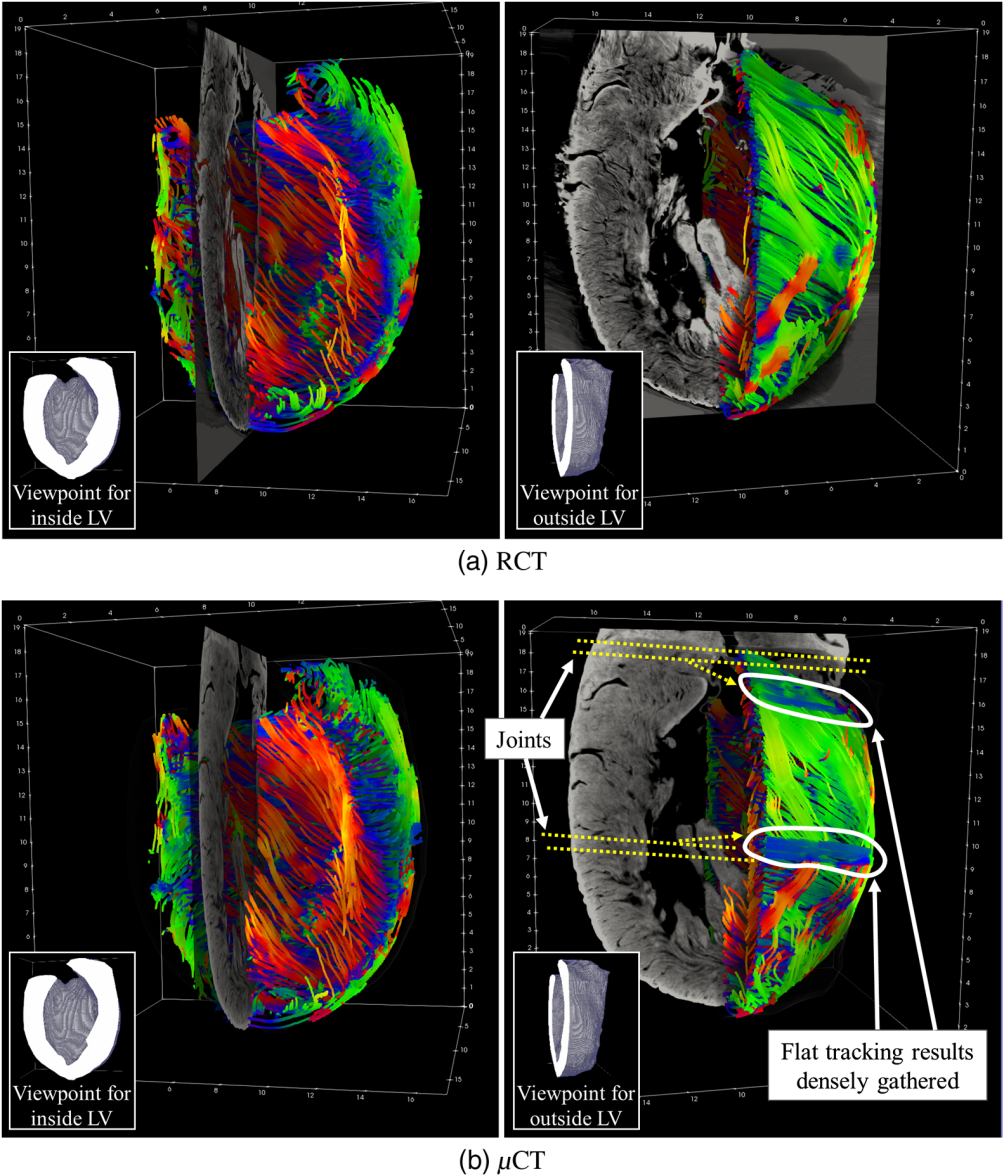
Fiber tracking results with sagittal slice. Colors represent inclination angles. Two viewpoints were defined: one for observing endocardium and another for epicardium. (a) RCT: tracking was performed properly in entire LV. (b) μCT: although closely resembling RCT results in (a), flat tracking results, densely gathered in joints, were produced due to scanning procedure, explained in Sec. 3.1.

Figure 11 shows the fiber trajectories cropped along the coronal plane and a sagittal slice of the μCT or RCT volumes. Colors showing the inclination angles are red inside the LV and green outside it. These color tendencies visually confirm the correspondence of the outside ratio and the inclination angles. However, from the μCT results [Fig. 11(b)], some fiber tracking results were flat and densely gathered. This tendency was not observed in the RCT results [Fig. 11(a)]. These incorrect tracking results from the μCT volume were caused by the joints produced by the scanning procedure, as explained in Sec. 3.1.

## 6 Discussion

### 6.1 Fiber Orientation Statistics

The μCT visually had lower contrast for the heart shown in Figs. 3 and 5. The fiber orientation estimations were not very similar to those of the RCT volume, which had an average error around 20 deg (Fig. 7). The average error values were increased by outliers, like the red bars in Fig. 8.

In the part magnified in Fig. 8, many outliers are observed. These errors were caused by an iodine solution artifact (having a higher absorption of x-ray) used for contrast enhancement of μCT imaging (Fig. 8). This iodine solution created a strong artifact in a slice plane. A tracking algorithm traced it and produced in-plane (flat) tracking.

The colors of the points in Fig. 9 suggest that the inclination angles computed from both the RCT and μCT volumes were positive inside and negative outside the LV. This tendency was already proved through anatomical studies,^5^ and the results of both the μCT and RCT volumes followed it.

We used nonrigid registration to compensate for the deformation of the specimen at the RCT and μCT scanning times. Our scanning procedures were performed in the following order: RCT scanning, iodine staining, and μCT scanning, as explained in Sec. 3.1. Iodine staining caused a slight contraction of the heart. There were some changes in the specimen sizes and small structures between the two CT volumes.

### 6.2 3-D Visualization of Fibers

Fiber tracking allows intuitive understanding of fiber running orientations in 3-D space. The tendency of inclination angles, correlated to the outside ratio, was also visually observed in the fiber tracking results from both the RCT and μCT volumes (Fig. 11). Most of the fiber tracking results inside the LV were red, and most of those outside were green. The trajectories were visually smooth from both volumes.

Figure 11 shows the fiber tracking results from the base to the apex. One large difference between the RCT and μCT volumes is apparent. On the results from the μCT volume [Fig. 11(b)], flat tracking results are densely gathered. Since our μCT scanner had a limited FOV, the rabbit heart was scanned by dividing it into three parts (Sec. 3.1). Since the images of the three scanning results were not precisely aligned, their joints were followed by tracking. Correction processes for such mistracking are required in the future.

We found that it is possible to estimate fiber orientation well on μCT volumes. Our fiber orientation estimation procedures were useful for fiber tracking in the entire LV, although the results must be scrutinized for errors between two scans. μCT, which is a promising modality for cardiac imaging and useful for observing cardiac fibers, is commonly used by many companies and institutes for industrial purposes. Our work shows an application for cardiac imaging, which presents imaging protocols and their usefulness for observing cardiac fibers.

## 7 Conclusions and Limitations

We described our fiber analysis methods from the RCT or μCT volumes of the heart and analyzed how μCT produces proper results using our methods by comparing them with RCT. A rabbit heart was fixated by ethanol, scanned by RCT, stained in an iodine solution, and scanned by μCT. The RCT and μCT volumes were nonlinearly registered. The fiber orientation of each point was estimated using the ST analysis of each volume. We defined two measures, the angle difference of μCT from RCT and inclination angles, to compare the fiber orientation estimation results at the sample points of these volumes. Although promising results were obtained in the cardiac fiber analysis using μCT, we need to perform further investigation on the differences of the results obtained from the μCT and RCT volumes. Analysis results from both modalities match well the anatomical knowledge that fiber orientations are different inside and outside of the LV. Unfortunately, the μCT volume caused incorrect tracking around the boundaries of the scanning of the stitching. Smoothing around the boundaries is our future work.

Our work suffers from the following limitations. First, just one specimen is inadequate for comparison experiments of two scans and their analysis. However, this problem is caused by restricted usage of RCT scanning that needs to use the synchrotron facility (circumference: 187 m) shared by worldwide high-energy physics researchers. Obtaining beam time is difficult and expensive. This is why we have only one sample. Increasing the number of samples is also future work. Second, the quantitative validation of fiber orientation results is required from a RCT volume that has been used as ground-truth. Several manually set parameters and the evaluation of different sets of parameters are also needed. One idea is a comparison with histopathological sections, but such a project is very complicated, as explained in Sec. 1. Therefore, future work will include deeper validation using many more hearts to quantitatively validate the fiber estimation of SRs for RCT volumes. We would also like to find ways to observe not only fibers in the LV but also other parts and tissues in the heart.
